# Epidemiology of acute rubella infection in Zambia during the pre-vaccination period (2005–2016) as a baseline for monitoring rubella epidemiology in the post-rubella vaccine introduction era

**DOI:** 10.1186/s12879-020-4806-5

**Published:** 2020-02-03

**Authors:** Mazyanga L. Mazaba, Seter Siziya, Mwaka Monze, Daniel Cohen

**Affiliations:** 1 The Health Press – Zambia, Zambia National Public Health Institute, Ministry of Health, Lusaka, Zambia; 2 Michael Chilufya Sata School of Medicine, Copperbelt University, Ndola, Zambia; 3 Virology Unit, University Teaching Hospital, Ministry of Health, Lusaka, Zambia; 4 Sackler Faculty of Medicine, Tel Aviv University, Tel Aviv, Israel

**Keywords:** Rubella, Acute, Proportion, Correlates, Zambia, Pre vaccination era, Immunisation

## Abstract

**Background:**

Rubella is highly under reported in Zambia as in most sub-Saharan countries despite being a disease of major public health concern especially among women of childbearing age. In September 2016, Zambia introduced a combined measles-rubella vaccine in children 0–14 years. In this study, we estimated the proportion positive for acute rubella among suspected but negative measles cases between 2005 and 2016 and determined its correlates for monitoring rubella epidemiology post-rubella vaccine introduction.

**Methods:**

In a retrospective study, 4497 measles IgM negative serum samples from 5686 clinically suspected measles cases were examined for rubella IgM antibodies using the Siemens, Enzygnost® ELISA kit at the national measles laboratory. Data on demographics, year and month of onset were extracted from the surveillance data. Multivariate logistic regression analysis using backward variable selection was conducted to determine independent predictors for acute rubella. The magnitude of association was estimated using adjusted odds ratio with a 95% confidence interval.

**Results:**

Overall, a proportion of 29.2% (1313/4497) affecting mostly those between 5 and 24 years was determined. Only age, province, month and year were independently associated with acute rubella. The regional proportions varied from 21.8–37.3% peaking in the month of October. Persons in the age group 10–14 years (Adjusted Odds Ratio [AOR] = 2.43; 95% CI [2.01–2.95]) were more likely while those aged < 1 year less likely (AOR = 0.31; 95% CI [021–0.48]) to have acute rubella compared to those aged 25 years or older. Persons in 2010 were less likely (AOR = 0.12; CI [0.05, 0.28]) to have acute rubella compared to those in 2016. While acute rubella was more likely to occur between July and November compared to December, it was less likely to occur between February and May.

**Conclusions:**

Rubella virus was circulating in Zambia between 2005 and 2016 affecting mostly persons in the age group 5–24 years peaking in the hot dry season month of October. Although vaccination against rubella has been launched, these baseline data are important to provide a reference point when determining the impact of the vaccination program implemented.

## Background

Rubella is highly under reported in Zambia as in most sub-Saharan countries, despite being a disease of major public health concern, more so amongst young women in childbearing age causing miscarriage, foetal death or an infant born with malformations [1]. Rubella infection is prevalent in Africa. In a recent review of literature, Goodson [2] reported rubella Immunoglobulin M (IgM) positivity rates among suspected measles cases ranging from 14 to 40% in the World Health Organization African Region between 2002 and 2009. Descriptive studies on measles surveillance programs in Africa indicate higher acute rubella positivity rates among the 5–9 years [2, 3] and 10–14 years age group [4, 5].

Various correlates for rubella include socio-demographic factors such as age, sex, year, season and region. Comparisons of infection rates between and within countries and different subpopulations may not be valid partly due to differences in criteria for rubella positivity that have varied from 1:8 to 1:40 [6]. Although results on the association of age with rubella infection have not been consistent, generally age has been reported to be significantly associated with rubella. While some studies revealed an association of acute rubella with age [7–9], Barreto et al. [10] did not find a significant association with rubella IgG positivity. Noting limited information on the association with sex, the proportion of rubella antibodies has been reported to be higher in females than males [6].

Seasonality has been associated with acute rubella. A study by Goodson et al. [2] analyzing the rubella epidemiology in Africa indicates the prevalence peaking in March–April in West sub-Saharan African; in February in the Central sub-Saharan African; in March–April and in September–October in East sub-Saharan African; and in September to October in South sub-Saharan African. Although higher rubella IgM positivity rates have been noted in the hot dry seasons, some variations have been recorded with the peak in West, Central and East Africa coinciding with the rain season [2, 11]. Rubella epidemics in the pre-vaccine era have been well documented to occur every 6–8 years (or 5–9 years) [12]. In a literature review, Goodson et al. [2] reported that rubella IgM positivity rate was higher in rural (63%) than urban (37%) settings. In another study, Mitiku et al., [13] also reported a higher acute rubella infection rate in urban (19.4%) than rural (11.6%) settings. However, to the contrary, Barreto et al. [10] found no significant difference in proportions of rubella IgG antibodies between rural and urban areas.

Zambia intensified laboratory-backed measles case based surveillance in 2003. During the period under review, Zambia had no programmatic goal towards elimination of rubella and neither did it have a vaccination policy against rubella infection. In October 2016, Zambia introduced a combined measles-rubella vaccine in children aged between 9 months and 14 years through a nationwide campaign. There is scanty evidence in Zambia on the epidemiology of rubella in the pre-vaccination period. The objective of the study was to determine the proportion and demographic correlates of acute rubella infection in Zambia before the introduction of mass measles-rubella immunization.

## Methods

### Study design

A retrospective analysis of data captured by the national measles laboratory through the national measles case based surveillance from January 2005 to September 2016 was conducted.

### Study population and setting

Cases among the laboratory investigated suspected measles cases in Zambia tested for rubella IgM in the virology laboratory at the University Teaching Hospital in Lusaka between January 2005 and September 2016.

### Case definition

A suspected measles case was defined as any person who presented with fever, generalized maculopapular rash, and either cough, or coryza, or conjunctivitis regardless of age and sex or any person whom a clinician suspected to have measles [14].

A case definition for rubella was rubella IgM positivity in a suspected measles case testing negative to measles IgM. IgM is the largest antibody, and it is the first antibody to appear in the response to initial exposure to an antigen [15].

### Laboratory analysis

Samples collected from suspected measles cases mostly within 14 days of onset of rash according to WHO/AFRO guidelines [16] that tested negative or equivocal to measles IgM were routinely tested for rubella IgM at the Zambia National Measles Laboratory (Virology laboratory, University Teaching Hospital in Lusaka) accredited by WHO. The serum samples were qualitatively analysed for rubella Immunoglobulin M (IgM) by the Enzyme Linked Immunosorbent Assay (ELISA) using the Siemens.Enzygnost® with specificity and sensitivity of 98.5 and 100%, respectively [17].

The difference in the optical densities (Absorbance) as stipulated in the manufacturer’s guidelines, ΔA between the antigen and antigen control wells was calculated and results interpreted as follows:

Anti-Rubella-Virus/IgM negative ΔA < 0.100.

Anti-Rubella-Virus/IgM positive ΔA > 0.200.

Anti-Rubella-Virus/IgM equivocal 0.100 ≤ ΔA ≤ 0.200.

### Data management and analysis

Data capture was done using Epi info version 3.5.4.9, software developed by the Center for Diseases Control and Prevention, Atlanta, USA. The data entry program had drop down options for some variables including sex, district and province to eliminate errors. Considering the variable date including date of birth and date of onset of rash with too many possibilities, was not locked on entry. Data cleaning was performed using frequencies and consistency checks to further minimize errors.

Among the variables extracted from the surveillance data for analysis were age, sex, province, year and month of onset. Multivariate Logistic regression using a backward variable selection method was conducted to determine independent predictors for acute rubella. The magnitude of association was estimated using adjusted odds ratio with a 95% confidence interval.

Still the odd typo - e.g. line 18 on the page with data management and analysis should say.

## Results

**Fig. 1:**
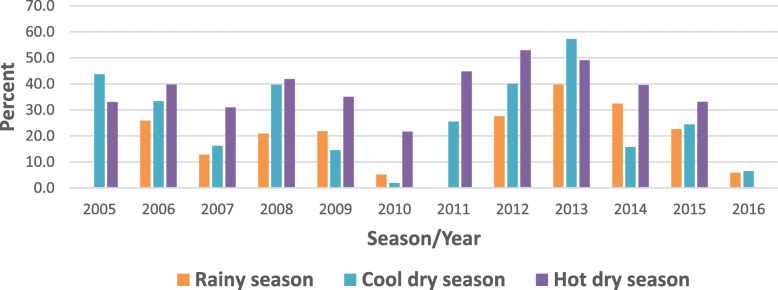
Seasonal trends by year of acute rubella prevalence (Zambia Jan 2005-Sep 2016)

**Fig. 2:**
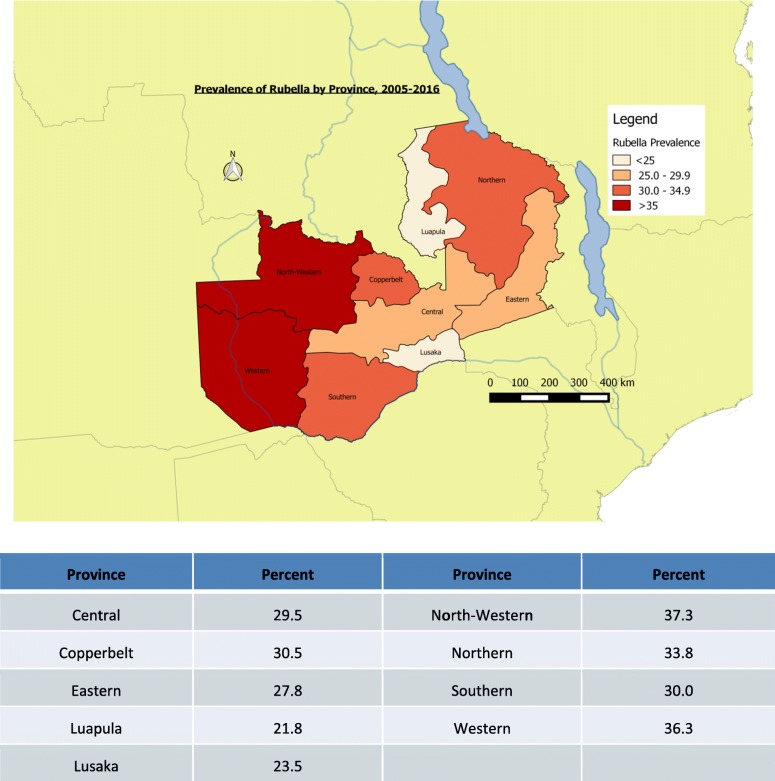
Prevalence of acute rubella by province (Zambia Jan 2005-Sep 2016)

Of the total 5683 suspected measles samples examined between 2005 and September 2016, 4497 measles negative and equivocal samples were tested for rubella IgM. The overall proportion of acute rubella was 29.2% (1313/4497). Acute rubella positivity among the females was 30.8 and 27.7% among males (*p* = 0.022). The age group 10–14 years had the highest proportion (41.8%) followed by the 5–9 years age group (35.9%), while the lowest (7.5%) was among infants. Among the years, the lowest positivity rate of acute rubella (4.5%) was in 2010 and the highest (49.4%) in 2013.The prevalence of acute rubella in Zambia started to peak in July through to November with the highest prevalence in October (Table 1). Generally, the largest proportion of acute rubella occurred in the hot dry season (Fig. 1). North-Western province recorded the highest proportion of acute rubella at 37.3% and the lowest was Luapula with a proportion of 22.8% (Fig. 2).

**Table 1:** Distribution of rubella IgM antibody levels by sex, age, month and year in measles-negative serum samples between 2005 and September 2016

Factor	Total tested	Rubella IgM		
		Positive	Negative	Equivocal
	n (%)	n (%)	n (%)	n (%)
Total sample	4497 (100)	1313 (29.2)	2928 (65.1)	256 (5.7)
Sex				
Male	2333 (100)	647 (27.7)	1545 (66.2)	141 (6.0)
Female	2160 (100)	666 (30.8)	1379 (63.8)	115 (5.3)
Age (years)				
< 1	254 (100)	19 (7.5)	230 (90.6)	5 (2.0)
1–4	1336 (100)	278 (20.8)	1001 (74.9)	57 (4.3)
5–9	1322 (100)	475 (35.9)	744 (56.3)	103 (7.8)
10–14	739 (100)	309 (41.8)	380 (51.4)	50 (6.8)
15–24	414 (100)	126 (30.4)	263 (63.5)	25 (6.0)
25+	114 (100)	14 (12.3)	94 (82.5)	6 (5.3)
Month				
January	238 (100)	52 (21.8)	178 (74.8)	8 (3.4)
February	300 (100)	43 (14.3)	248 (82.7)	9 (3.0)
March	342 (100)	57 (16.7)	273 (79.8)	12 (3.5)
April	282 (100)	37 (13.1)	235 (83.3)	10 (3.5)
May	310 (100)	27 (8.7)	268 (86.5)	15 (4.8)
June	307 (100)	65 (21.2)	229 (74.6)	13 (4.2)
July	435 (100)	169 (38.9)	240 (55.2)	26 (6.0)
August	432 (100)	169 (39.1)	233 (53.9)	30 (6.9)
September	490 (100)	166 (33.9)	297 (60.6)	27 (5.5)
October	552 (100)	244 (44.2)	263 (47.6)	45 (8.2)
November	528 (100)	204 (38.6)	279 (52.8)	45 (8.5)
December	281 (100)	80 (28.5)	185 (65.8)	16 (5.7)
Year				
2005	580 (100)	190 (32.8)	342 (59.0)	48 (8.3)
2006	237 (100)	72 (30.4)	151 (63.7)	14 (5.9)
2007	425 (100)	101 (23.8)	299 (70.4)	25 (5.9)
2008	731 (100)	255 (34.9)	421 (57.6)	55 (7.5)
2009	316 (100)	76 (24.1)	239 (75.6)	1 (0.3)
2010	357 (100)	16 (4.5)	334 (93.6)	7 (2.0)
2011	175 (100)	62 (35.5)	99 (56.6)	14 (8.0)
2012	341 (100)	150 (44.0)	165 (48.4)	26 (7.6)
2013	358 (100)	177 (49.4)	147 (41.1)	34 (9.5)
2014	284 (100)	87 (30.6)	187 (65.8)	10 (3.5)
2015	394 (100)	98 (24.9)	281 (71.3)	15 (3.8)
2016	299 (100)	29 (9.7)	263 (88.0)	7 (2.3)

Sex was not independently associated with acute rubella. Age, province, month and year were independently associated with acute rubella. Persons in the age group 10–14 years (Adjusted odds ratio [AOR] = 2.43; 95% CI [2.01–2.95]) were more likely and those aged < 1 year (AOR = 0.31; 95% CI [021–0.48]) were less likely to have acute rubella compared to those aged 25 years or older. Persons in 2010 were less likely (AOR = 0.12; CI [0.05, 0.28]) to have acute rubella compared to those in 2016. Acute rubella was more likely to occur between July and November (AOR = 1.66; CI [1.33, 2.08], AOR = 1.86; CI [1.49, 2.32], AOR = 1.29; CI [1.03, 1.60], AOR = 2.15; [1.77, 2.62] and AOR = 1.60; CI [1.31, 1.95], respectively) compared to December, and less likely to occur between February and May (AOR = 0.52; CI [0.37, 0.72], AOR = 0.48; CI [0.36, 0.65], AOR = 0.62; CI [0.43, 0.88] and AOR = 0.44; CI [0.30, 0.65] respectively) as shown in Table 2.

**Table 2:** Independent factors associated with acute rubella (Zambia 2005–2016)

Factor	Adjusted Odds Ratio (95% CI)
Age (years)	
< 1	0.31 (0.21–0.48)
1–4	0.98 (0.82–1.17)
5–9	1.97 (1.66–2.34)
10–14	2.43 (2.01–2.95)
15–24	1.34 (1.06–1.69)
25+	1
Province	
Central	1.00 (0.79–1.27)
Copperbelt	0.91 (0.76–1.08)
Eastern	1.10 (0.82–1.49)
Luapula	0.63 (0.48–0.83)
Lusaka	0.84 (0.71–0.99)
North-Western	1.40 (1.05–1.85)
Northern	1.16 (0.92–1.45)
Southern	0.93 (0.76–1.13)
Western	1
Month	
January	1.04 (0.75–1.43)
February	0.52 (0.37–0.72)
March	0.48 (0.36–0.65)
April	0.62 (0.43–0.88)
May	0.44 (0.30–0.65)
June	0.88 (0.66–1.18)
July	1.66 (1.33–2.07)
August	1.86 (1.49–2.32)
September	1.29 (1.03–1.60)
October	2.15 (1.77–2.62)
November	1.60 (1.31–1.95)
December	1
Year	
2005	1.43 (1.16–1.75)
2006	1.06 (0.78–1.43)
2007	0.93 (0.73–1.19)
2008	1.68 (1.39–2.04)
2009	1.00 (0.74–1.36)
2010	0.12 (0.05–0.28)
2011	1.32 (0.91–1.91)
2012	1.95 (1.49–2.56)
2013	2.53 (1.99–3.23)
2014	1.50 (1.14–1.98)
2015	0.87 (0.68–1.12)
2016	1

## Discussion

A 29.2% proportion of acute rubella was found among clinically suspected measles cases investigated through the national laboratory backed measles case based surveillance program between 2005 and 2016 in Zambia. The prevalence of acute rubella amongst females was 30.8 and 27.7% among males, although sex was not independently associated with acute rubella. Only age, province, month and year were independently associated with acute rubella.

The proportion of acute rubella cases in the current study and in similar studies is based on those who had clinical symptoms that met the measles case definition. It is known that rubella infection may be subclinical in up-to 50% of rubella infections [18], therefore this method of identifying infections will not include all rubella infections. The proportion of acute rubella in the present study is similar to what has been reported in other parts of Africa of 14–40% in the pre-vaccination period 2002–2009 [2]. The findings from the current study indicate that Zambia has a lower overall prevalence rate of acute rubella compared with more recent data from Zimbabwe (37.6%) [4] and Ethiopia (39.4%) [13] but higher than Cameroon (9.3%) [3] and comparable to Central African Republic (30.2%) [5]. The varying prevalence rate for acute rubella indicates the different transmission patterns among countries [19] and variations in climatic conditions [20].

The current study results indicate that persons in the age group 5–24 years were more likely to have acute rubella compared those aged 25 years or older. Meanwhile persons aged < 1 year were less likely to have acute rubella compared to persons aged 25 years or older. Persons under the age of one year are still protected by maternal antibodies and the older they become they lose this protection. Similar findings of a significant association between age and rubella have been reported before [2].

**Fig. 3:**
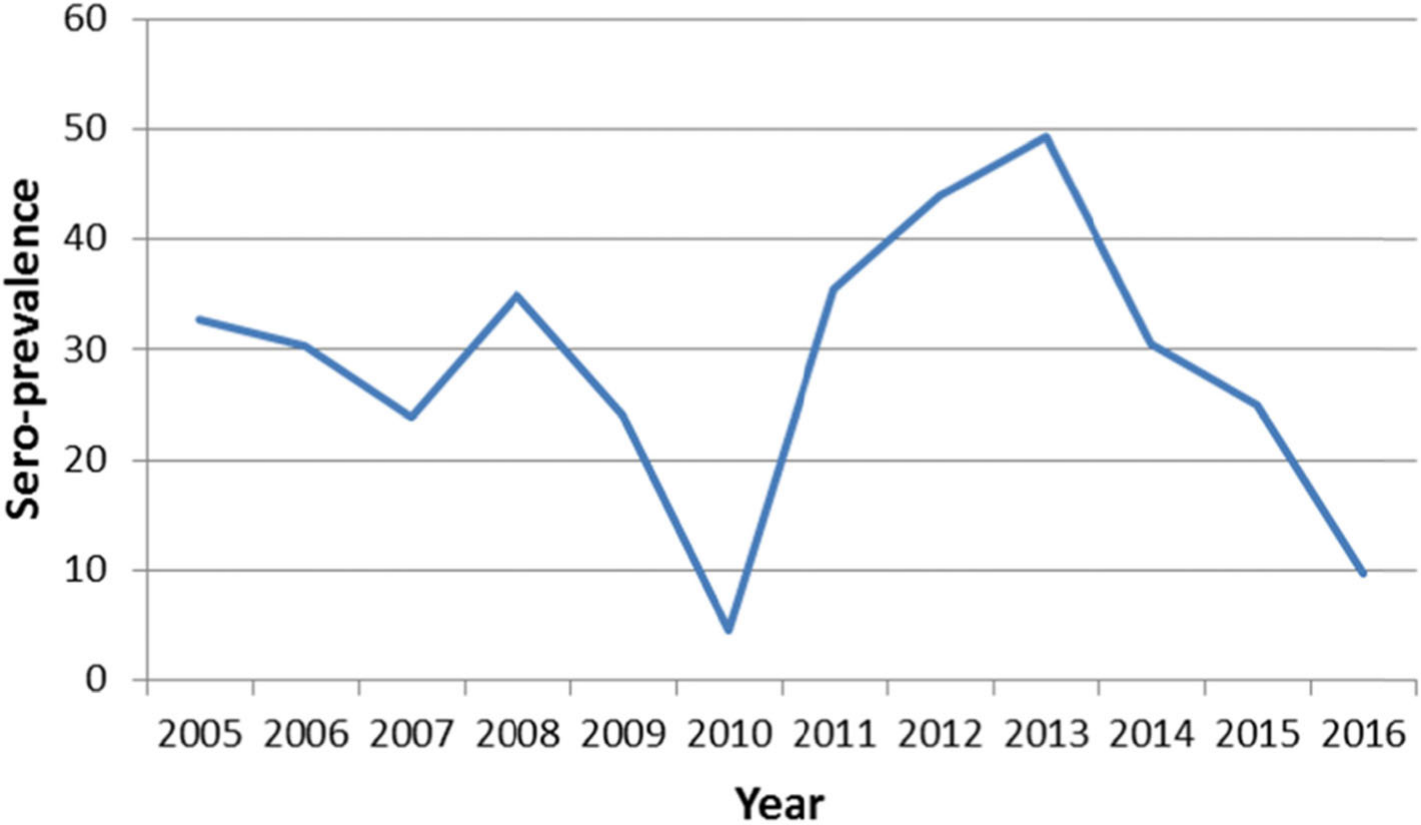
Acute rubella prevalence by year (Zambia 2005–2016)

The prevalence of acute rubella in the present study peaked in October (Fig. 3), the hottest month. Elsewhere in west, central and east Africa, the peak for acute rubella coincides with the rainy season. The seasonality of acute rubella in the southern Africa region has not been consistent. The current finding is in agreement with the observation that acute rubella in the southern Africa region generally coincides with the hot dry season [2]. The finding from the current study accords what has been reported in Zimbabwe that acute rubella peaks in October–November just before the start of rainy season [4]. Further investigations are warranted to determine climatic factors associated with rubella virus in the southern region of Africa that has varying climatic conditions from the Mediterranean climate climatic in Cape Town, South Africa, desert conditions in Namibia to tropical/sub-tropical climate in Zambia; and in particular a study on association between climate and rubella infection would be interesting in Zambia since although Zambia has tropical climate generally, the climate is modified by altitude in different regions of the country.

The acute rubella epidemic interval in the current study was not clearly defined. However, findings elsewhere suggest l0-year intervals [11] and 5–9 years [12] for rubella infection. There is need to gather more data points when epidemics occur in Zambia in order to accrue more evidence for predicting epidemics.

The findings that the prevalence of acute rubella was lowest in Lusaka and Luapula provinces and highest in Western and North-western provinces is not entirely clear. It may be speculated that the herd immunity to acute rubella may be higher in Lusaka due to continuous outbreaks over the years. Differences in elevations of the provinces and climatic conditions may explain variations in acute rubella by province.

### Limitations

It is possible that the proportion of acute rubella among clinically suspected measles cases who were measles IgM negative was underestimated because rubella being generally a milder fever/rash illness a substantial number of rubella cases may not have entered the reporting system to qualify for rubella IgM testing. The kit that was selected had high specificity and sensitivity of 98.5 and 100%, respectively [17], indicating that cross-reactions with other diseases were a minimum.

## Conclusion

There is evidence that rubella virus was circulating in Zambia between 2005 and 2016 affecting persons in the age group 5–24 years, with a peak in the hot dry season. Although vaccination against rubella has been launched, these baseline data are important to provide a reference point when determining the impact of the vaccination program implemented. There is need to understand further why acute rubella positivity is lower in Luapula compared to other areas of similar rural settings. It is recommended that immunisation campaigns be targeted to those aged 5–24 years in a situation of limited resources followed by routine immunisation.

## Data Availability

Data is owned by Ministry of Health and permission to utilise it was sought and approved. Only parameters utilised in this paper can be accessed.

## Abbreviations

AFRO: Africa Regional Office
AOR: Adjusted Odds Ratio
CI: Confidence Interval
IgG: Immunoglobulin G
IgM: Immunoglobulin M
WHO: World Health Organisation
